# Case Report: Pyoderma gangrenosum misdiagnosed as diabetic foot

**DOI:** 10.3389/fendo.2025.1604157

**Published:** 2025-08-29

**Authors:** Juyun Zhang, Jicong Jiang, Meimei Wu, Qing Xiong

**Affiliations:** ^1^ Department of Endocrinology, Haikou Affiliated Hospital of Central South University Xiangya School of Medicine, Haikou, China; ^2^ Department of Healthcare, Haikou Affiliated Hospital of Central South University Xiangya School of Medicine, Haikou, China

**Keywords:** pyoderma gangrenosum, glucocorticoid, case report, diabetic foot, ulcer

## Abstract

Pyoderma gangrenosum (PG) is a rare neutrophilic dermatosis characterized by pain and sterile ulcers. PG in diabetic patients is uncommon and often misdiagnosed as diabetic foot. This paper reports a case of a diabetic patient with a painful foot ulcer, ultimately diagnosed as PG.

## Introduction

Pyoderma gangrenosum (PG) is a rare non-infectious, ulcerative neutrophilic dermatosis triggered by neutrophil dysfunction. It is characterized by painful, sterile ulcers, often located on the lower extremities and trunk, and is associated with inflammatory reactive dermatoses that lead to skin destruction (1, 2). The pathogenesis of PG remains unclear, but it is currently believed to involve dysregulation of the immune system, neutrophil dysfunction, genetic susceptibility, and abnormal inflammation (3, 4). PG is frequently comorbid with underlying systemic conditions such as inflammatory bowel disease (IBD), rheumatic disorders, hematological disorders, and malignancies (5, 6). PG in patients with diabetes mellitus is rare and can easily be misdiagnosed as diabetic foot, which can impact treatment and prognosis. The clinical data of a patient admitted to our hospital are presented and discussed below.

## Case

**Figure 1:**
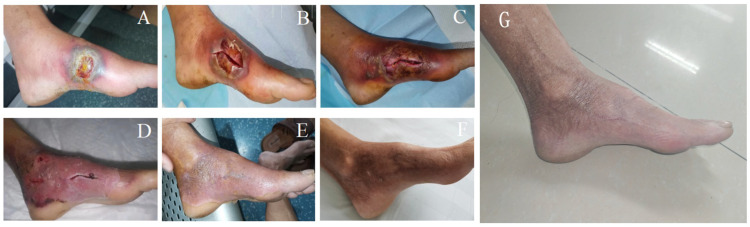
Foot ulcer of the patient. **(A)** On the admission; **(B)** The day after admission; **(C)**: On the fourth day after admission; **(D)** After 4 days of methylprednisolone treatment; **(E)** After 2 weeks of methylprednisolone treatment; **(F)** Check after 3 months; **(G)** A follow-up visit in February 2025.

**Figure 2:**
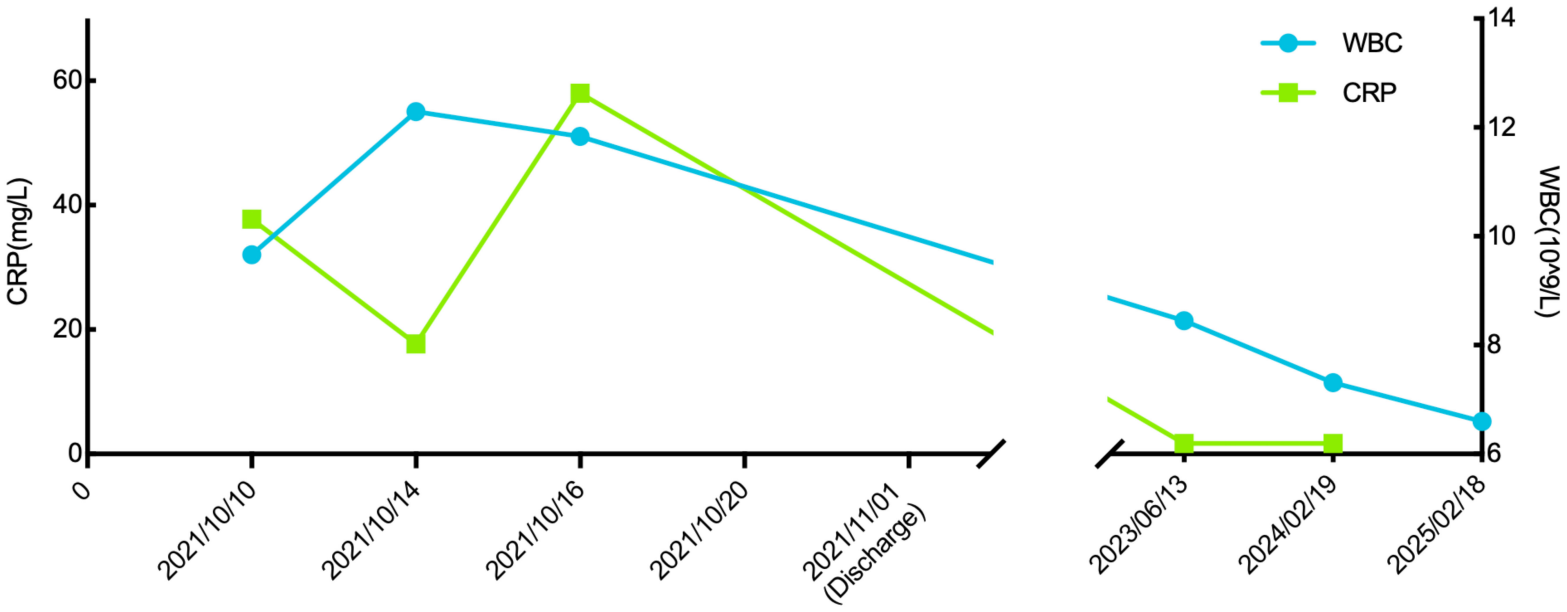
Changes in inflammatory markers during the patient’s hospitalization.

**Figure 3:**
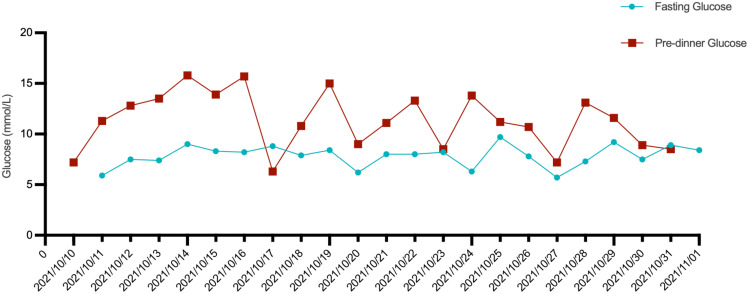
The patient’s fasting blood glucose and pre-dinner blood glucose levels during hospitalization, as well as the antidiabetic medications used.

**Figure 4:**
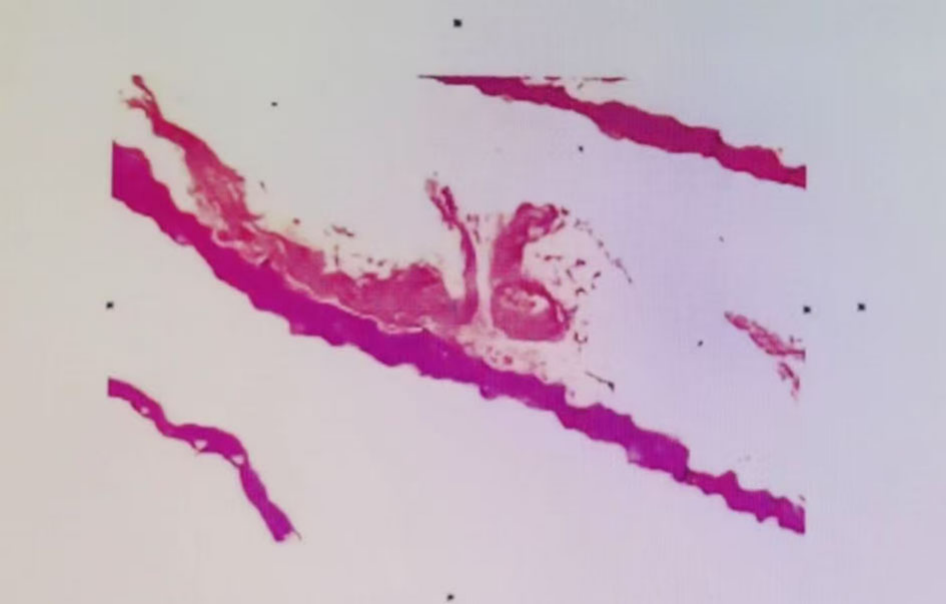
The patient’s pathological tissue results show the stratum corneum to the spinous layer, with degeneration and necrosis, along with a small amount of hemorrhagic necrosis. The pathological diagnosis is a small amount of degenerative and necrotic skin tissue on the left ankle.

A 57-year-old male patient presented to the endocrinology department with dry mouth, excessive drinking for two months, and a newly developed ulcer on his left foot for one week. At the time of admission, he had been diagnosed with type 2 diabetes(T2DM) for two months but had not been taking any hypoglycemic agents. His fasting terminal blood glucose level was approximately 9.0 mmol/L. History inquiry revealed one red, inflamed, sore but not itching nodules measuring as the size of a grain of rice on his left foot one week prior to admission. After one day, the nodules began to rupture, the pain increased, and the ulcers and redness centered at the nodules gradually expanded, with a little oozing. The patient denied any drug allergies, and there was no family history of similar diseases. The initial clinical examination revealed a 1 cm × 1 cm ulcer on the medial side of the left foot, with surrounding erythema extending 4 cm × 4 cm. The erythema had well-defined borders, some areas appearing purplish-red. No obvious fluctuation was noted, but local skin temperature was elevated, and tenderness was significant. The patient’s blood routine showed: leukocytes at 9.66×10^9^/L, neutrophil ratio at 64.3%, neutrophil count at 6.21×10^9^/L; C-reactive protein at 37.79 mg/L, erythrocyte sedimentation rate at 33.55 mm/h, other blood tests showed glycated hemoglobin at 8.90%. Thyroid and adrenal function tests were within normal ranges. Serum calcitonin was also unremarkable. A vascular ultrasound of this patient showed no significant abnormalities. X-rays of the left foot and ankle showed the presence of degenerative changes in the patient’s left ankle joint. The patient’s electromyography indicated abnormal sympathetic reaction of both lower limbs. The patient was diagnosed as type 2 diabetic foot ulcer and infection. He underwent an incision and decompression, debridement, and regular wound dressing changes. Simultaneously, he was started on hypoglycemic therapy, antibiotics, and symptomatic treatment. However, despite these treatments, the ulcer and surrounding erythema continued to expand progressively, with worsening pain. The affected skin became purplish-red, partially necrotic, and the infection spread rapidly. Given the worsening infection, the patient was switched to meropenem and vancomycin for seven days, but the condition continued to deteriorate. The redness and swelling extended to the ankle joint and bunions, with increased wound exudation. Repeating blood routine showed that leukocytes at 11.84×10^9^/L, neutrophil ratio at 71.9%, neutrophil count at 8.52×109/L, C-reactive protein at 58 mg/L; erythrocyte sedimentation rate at 64.87 mm/h. Multiple cultures of wound secretions were taken indicating no bacterial growth. As necrotizing fasciitis could not be ruled out in the surgical consultation, debridement and closed negative pressure wound therapy (NPWT) were given. However, the ulcer continued to expand. After two days of NPWT, the ulcer spread above the ankle joint, accompanied with severe pain. A histopathological biopsy revealed degenerative necrosis extending from the stratum corneum to the stratum spinosum. After a comprehensive reevaluation, pyoderma gangrenosum (PG) was considered as a potential diagnosis. The subsequent pathology report of the wound tissue biopsy reveals a small sample of skin tissue, showing changes from the stratum corneum to the stratum spinosum, accompanied by degeneration and necrosis, with a small amount of hemorrhagic necrosis observed. Based on the patient’s pathological report, we began to suspect that the ulcer was non-infectious and thus initiated glucocorticoid therapy. Then the patient was treated with methylprednisolone tablets 16mg twice daily orally and silver ion gel (every other day) on the wound. Following the initiation of methylprednisolone and silver ion gel, the patient experienced significant relief of pain and swelling. By the next day, ulcer progression halted, and gradual improvement was observed. After this, we reduced the methylprednisolone tablets to 12mg bid, at which time the patient’s redness, swelling and pain improved obviously. and the methylprednisolone tablets were gradually tapered to discontinuation. Therefore, we gradually reduced the dose of methylprednisolone tablets to the point of discontinuation. After 4 weeks of corticosteroids therapy (from October 20 to November 16), the patient’s wounds fully healed. Although the patient’s pathological report did not show classical findings of pyoderma gangrenosum (PG), the patient could still be diagnosed with PG according to the Paracelsus diagnostic criteria proposed by Jockenhöfer et al. in 7. In the Paracelsus score, this patient scored 13 points, meeting the major criteria of “Progressing disease” and “Reddish-violaceous wound border”; the minor criteria of “Amelioration by immunosuppressant drugs”, “Characteristically irregular (bizarre) ulcer shape”, “Extreme pain >4/10 on visual analogue scale” and “Localization of lesion at site of trauma”; as well as the additional criterion of “Undermined wound border”(Please refer to **Table 1**). A score of 10 or higher indicates a high suspicion of PG. The changes in the patient's foot ulcer after treatment are shown in **Figure 1**. The changes in the patient's inflammatory markers and the start time of hormone use are shown in **Figure 2**. The changes in the patient's blood glucose levels during hospitalization and the use of antidiabetic drugs are shown in **Figure 3**. **Figure 4** shows the pathological results of the patient's ulcer tissue.

**Table 1:** Assessment of patients with suspected pyoderma gangrenosum (PG) using the PARACELSUS score. (Points ≥ 10, PG highly likely; points < 10, PG unlikely.).

Criteria	Points (for each one present)
Major criteria	3
Progressing disease Assessment of differential diagnoses Reddish-violaceous wound border	
Minor criteria	2
Amelioration by immunosuppressant drugs Characteristically irregular (bizarre) ulcer shape Extreme pain >4/10 on visual analogue scale Localization of lesion at site of trauma	
Additional criteria	1
Suppurative inflammation in histopathology Undermined wound border Systemic disease associated	

## Discussion

Pyoderma gangrenosum is a rare reactive non-infectious inflammatory disease which is characterized by painful, rapidly evolving cutaneous ulcers with undermined borders and peripheral erythema (8, 9). Several clinical variants of PG, including ulcerative, bullous, pustular, vegetative, peristomal and postoperative types. Among these, the ulcerative type is the most common (9). Diagnosing PG is challenging due to its variable clinical presentation, frequent association with underlying systemic diseases-such as inflammatory bowel disease (IBD), rheumatic diseases, hematologic disorders, and malignancies-and the lack of pathological evidence (5, 6). Due to the absence of specific biomarkers, PG is frequently misdiagnosed. Clinically, PG often begins as erythematous, edematous papules or sterile pustules, which later evolve into sharply marginated ulcers that are exceedingly painful, with raised, dark red or purple margins, submerged necrosis, and necrotic tissue or purulent exudate at the base (10). The lesion ultimately heals with an atrophic sieve-shaped scar (10).

The pathogenesis of PG remains unclear, though it is believed to involve neutrophil dysfunction, inflammatory cytokine dysregulation, and genetic predisposition (11). Additionally, allergic reactions and trauma are important triggers of PG (10).

Due to the lack of distinct serologic or histologic features and its non-specific clinical presentation, PG is often misdiagnosed as an infectious process or vasculitis (12). However, antibiotics and surgical debridement are ineffective for PG, and misdiagnosis can delay appropriate treatment. Inappropriate surgical interventions may exacerbate the condition, leading to further ulcer expansion. In some cases, multiple extensive debridements have failed, ultimately requiring amputation due to the progressive worsening of the disease (12). Therefore, prompt recognition and appropriate management of PG are crucial to prevent unnecessary harm to the patient. Currently, the diagnosis of PG relies on exclusion of infection, or whether hormone or immunosuppressive therapy is effective (5, 13). Systemic corticosteroids (CS), immunosuppressive agents, and biologic therapies are the main treatment options for PG. Among them, CS are considered first-line therapy due to their potent anti-inflammatory effects. A systemic CS dose of 0.5–1 mg/kg/day typically leads to clinical improvement within one week in most patients (10, 11). Moreover, cyclosporine as a calcineurin inhibitor, can also be used as a first-line drug for PG treatment by hampering the synthesis of ILs (11). Growing evidence supports the use of TNF-α inhibitors as first-line therapy, particularly infliximab. Anti-TNF-α agents, especially infliximab are the optimal choice for cases refractory to systemic glucocorticoids, cyclosporine, or their combination. They can also be used as conservative agents to avoid the long-term side effects of the aforementioned drugs. Other medications such as methotrexate, mycophenolate mofetil, azathioprine, systemic tacrolimus, dapsone, colchicine, thalidomide, and intravenous immunoglobulin have also been proven effective in treating pyoderma gangrenosum (PG). However, due to their adverse effects, they are generally not considered first-line treatments for PG (11).

Upon reviewing the diagnosis and treatment process for this case, it was initially misdiagnosed as diabetic foot with infection due to the patient’s history of diabetes mellitus, elevated blood glucose at the time of admission, local signs of redness, swelling, heat, and pain, as well as purulent secretion in the left foot, along with elevated C-reactive protein and erythrocyte sedimentation rate. The patient was treated with anti-infection therapy, incision and drainage, debridement, and dressing changes. However, the ulcer continued to expand, and the patient’s pain, redness, and swelling worsened, with the condition progressing rapidly. Repeated wound cultures showed no pathogenic bacteria, and histopathological examination revealed no specific changes, which was consistent with the clinical diagnosis of pyoderma gangrenosum (PG). Glucocorticoid therapy proved effective, supporting the diagnosis of PG. Three months later, the patient returned for follow-up, with the wound fully healed and no recurrence, leaving an atrophic, sieve-like scar.

## Conclusion

Diabetic patients are prone to foot infections and ulcers, a condition known as diabetic foot, due to complications such as neuropathy, vascular disease, and impaired immune defense, making it a major cause of disability and death. Pyoderma gangrenosum (PG) is much rarer, and when it occurs on the foot, it is even less common, often leading to misdiagnosis as diabetic foot. Similar cases have been reported before, but it is rarely discussed in China (14). In this case, the patient had a relatively short history of diabetes, with no significant peripheral neuropathy or vascular changes, and the rapidly progressing painful ulcer raised the suspicion of other possible causes for the ulcer. Both the PARACELSUS score proposed by Jockenhöfer F, Wollina U, et al., and the PG diagnostic criteria put forward by the Delphi Consensus of International Experts include inflammatory infiltration of the ulcer edge tissue as a diagnostic criterion. However, the tissue pathology of this patient does not show this feature, which adds to the difficulty in diagnosing this case (7).

Since diabetic foot and PG require vastly different treatments, misdiagnosis can lead to inappropriate management and disease progression. Diabetic foot is often complicated by bacterial infections, necessitating antibiotic therapy and surgical debridement. However, such interventions can worsen PG, leading to rapid disease progression. Therefore, early differentiation between diabetic foot and PG is critical. This case underscores the importance of considering PG as a differential diagnosis when a diabetic patient presents with an ulcer that does not respond to conventional treatment. Lack of infection and failure to improve with antibiotics should prompt suspicion of PG, allowing for timely initiation of immunosuppressive therapy and improved patient outcomes.

## Data Availability

The original contributions presented in the study are included in the article/supplementary material. Further inquiries can be directed to the corresponding author.
